# HMMR promotes peritoneal implantation of gastric cancer by increasing cell–cell interactions

**DOI:** 10.1007/s12672-022-00543-9

**Published:** 2022-08-24

**Authors:** Muwen Yang, Boyu Chen, Lingzhi Kong, Xiangfu Chen, Ying Ouyang, Jiewen Bai, Donglin Yu, Huizhong Zhang, Xinghua Li, Dongsheng Zhang

**Affiliations:** 1 Department of Experimental Research, State Key Laboratory of Oncology in South China, Collaborative Innovation Center for Cancer Medicine, Sun Yat-sen University Cancer Center, Guangzhou, 510060 Guangdong China; 2 Research Unit of Precision Diagnosis and Treatment for Gastrointestinal Cancer, Chinese Academy of Medical Sciences, Guangzhou, 510060 People’s Republic of China; 3 Academy of Traditional Chinese and Western Medicine, Binzhou Medical University, Yantai, China; 4 Department of Radiotherapy, Yantai Yuhuangding Hospital, Yantai, Shandong China

**Keywords:** Gastric cancer, HMMR, HA, Metastasis, Cell–cell interaction

## Abstract

**Background:**

Distant metastasis is the prominent factor for cancer-induced death of gastric cancer in which peritoneum is one of the dominating targets of gastric cancer metastasis. However, there is still a lack of effective predictive indicators and treatment methods for gastric cancer patients with peritoneal metastasis.

**Methods:**

A clustering assay was used to investigate the cell aggregates formation ability. While the soft agar assay and anoikis assay were performed to detect the anchorage-independent growth and anoikis-resistant ability respectively. Luciferase activity assay, western blotting and immunofluorescence were used to explore the effect of HMMR on AKT signaling activity. The peritoneal implantation model was examined to explore the role of HMMR in vivo.

**Results:**

Silencing of HMMR expression markedly reduced the peritoneal metastasis of gastric cancer cells through reducing cell–cell interactions. Mechanistically, HA-HMMR could activate Akt signaling, thus succeeding in distant colonization and metastatic outgrowth. Importantly, inducible depletion of HMMR significantly abrogates peritoneal implantation of gastric cancer in vitro and in vivo.

**Conclusion:**

Our study highlights that HMMR promotes peritoneal implantation of gastric cancer. A better understanding of HMMR’s functions and mechanism might provide a novel therapeutic target and prognostic marker for metastatic gastric cancer.

**Supplementary Information:**

The online version contains supplementary material available at 10.1007/s12672-022-00543-9.

## Introduction

Gastric cancer is one of the most common malignancies worldwide with high incidence and mortality, especially in China. In 2013, 427,100 new cases were diagnosed and 301,200 patients died of this disease in China [1, 2]. Distant metastasis is still a prominent factor for cancer-induced death of gastric cancer in which peritoneum is one of the distant dominating targets of gastric cancer metastasis [3, 4]. The median survival time of gastric cancer patients with distant metastasis was less than 3 months, especially with peritoneal dissemination [5, 6]. Thus, peritoneal metastasis, which lacks effective predictive indicators and treatment methods, remains the top priority in the diagnosis and treatment of this disease. However, little is currently known about the detailed molecular mechanisms of peritoneal metastasis in gastric cancer.

Peritoneal metastasis is thought to be a multistep process, in which tumor cells invaded and penetrated into the peritoneal cavity, then the exfoliated cells anchorage-independently grew and proliferated in the peritoneum [7, 8]. In the absence of anchorage, the detached cells need to develop adaptive strategies to prevent anoikis, such as formation of cell aggregation and cluster [9]. This anchorage-independent growth ability is essential for distant colonization and metastatic outgrowth [10, 11]. Recent work has proved that the clustering formation of cancer cells enhances its anoikis-resistant ability, thus protecting them from being rapidly cleared by NK cells and markedly increasing their metastatic potential [9, 12]. In addition, the multicellular aggregates of exfoliated gastric cancer cells expressed a stem cell-like phenotype, enhancing its proliferation in the abdominal cavity than scattered-free cancer cells [12, 13]. Clusters of cancer cells have been reported in various types of carcinomas encompassing gastric cancer [13–16]. Along with the cell clustering formation, increased cell–cell interaction, especially the interactions with components of the extracellular matrix (ECM) plays a vital role in distant metastatic outgrowth. Previous studies confirmed the interaction of cancer cells with ECM or peritoneum is crucial for its peritoneal dissemination [17–19]. Taken together, increased cell–cell interactions could promote the distant seeding and metastasis of cancer cells.

Hyaluronic acid (HA) is a kind of glycosaminoglycan widespread in the ECM [20]. Studies have found that HA levels are significantly increased in a variety of tumors and exhibited as a tumor marker and potential therapeutic target [21, 22]. Consistently, Hyaluronic acid synthases (HAS) promoting the synthesis and secretion of HA were also found to be generally highly expressed in tumors. CD44 and hyaluronan-mediated motility receptor (HMMR) are currently known as receptors of HA. Interaction between HA and its receptors, CD44 and HMMR, participates throughout the course of cancer progression and metastasis including cancer cell proliferation, adhesion, migration, differentiation and stem cell maintenance, thus playing an important role in tumor development and metastasis [23–25]. HMMR has been found markedly upregulated in colorectal cancer promoting growth, invasiveness, and dissemination of colorectal cancer [26]. As in gastric cancer, our previous research showed overexpression of HMMR caused chemotherapy resistance while silencing the expression of HMMR effectively increased the susceptibility to chemotherapeutic drugs [27]. However, the role of HMMR in peritoneal metastasis of gastric cancer remains obscure. Therefore, it is of great clinical significance to reveal the potential function and detailed mechanisms of HMMR in this metastatic course.

In this study, we demonstrated that HA-HMMR signaling promotes the anchorage-independent colony formation and enhances the anoikis-resistant ability of gastric cancer cells, thus succeeding in peritoneal dissemination and metastatic outgrowth. Mechanistically, HA-HMMR signaling activates AKT signaling, thus endowing the gastric cancer cells with metastatic capacity. Our results demonstrated HMMR might become a predictor and potential therapeutic target in gastric cancer patients with peritoneal metastasis.

## Materials and methods

### Cell culture

MKN28 and AGS cell lines were purchased from ATCC. Cell lines were authenticated by short tandem repeat finger printing. Cells were grown in Dulbecco’s Modified Eagle Medium supplemented with 10% fetal bovine serum, penicillin/streptomycin, hydrocortisone, insulin, HEPES, and L-glutamine.

### Plasmids and stable cell lines

To silence endogenous HMMR or HAS1 expression, the RNA oligonucleotides targeting HMMR or HAS1 were cloned into a pSuper-Retro-Puro Vector respectively. The shRNA sequences were provided in Supplementary materials and methods. Stable cell lines silencing HMMR or HAS1 were selected over 15 days culture using 0. 5 ug/ml puromycin.

### Western blot analysis

Protein was obtained using sample buffer containing phosphatase Inhibitor Cocktail (Cell Signaling Technology, Danvers, MA, USA). 10% SDS-PAGE was used to separated protein samples with the same amount (30 ug). The protein samples were then transferred into PVDF membranes. The PVDF membranes were blocked with 5% milk and then incubated with primary antibody at 4 °C, then the secondary antibody. Unprocessed scans of immunoblots are provided as Source Data in Additional file 2. The antibodies used in this study were as follows:

anti-HMMR rabbit polyclonal antibody (Sigma-Aldrich); anti-HAS1 rabbit polyclonal antibody (Sigma-Aldrich); anti-p-AKT, anti-AKT, anti-p-FOXO1, anti-FOXO1, anti-BAD, anti-BAX antibodies (Cell Signaling Technology), anti-GAPDH mouse monoclonal antibody (Sigma-Aldrich).

### Clustering assay

2.0 × 10^4^ gastric cancer cells were cultured in 1 ml of complete medium in a 15 ml conical tube at room temperature. The tubes were gently flicked during the process to visually assess the clusters progression of AGS and MKN28 cells. After 1–1.5 h of incubation, the cell suspension was poured into the wells of 12-well dishes and allowed to attach to the bottom of the dish for 5–10 min. Cells were promptly visualized and photographed under phase contrast and fluorescent filters using an AxioVert inverted microscope.

### Anchorage-independent growth ability assay

As 1 ml of complete medium with 1.3% agarose were plated as a bottom agarose layer, gastric cancer cells (n = 3000) digested using trypsin and suspended in 1 ml of complete medium with 0.6% agarose (Sigma) were plated on 6-well plates. Viable colonies were counted after 3 weeks of growth. The experiment was conducted three times independently. For each assay, an average of three replicates ± SD is shown.

### Anoikis resistance assay

For flow cytometry-quantified anoikis resistance assays, 2 × 10^4^ cells were seeded in serum-free media in six-well ultra-low attachment plates to observe anoikis. MKN28 and AGS cells were then incubated for 10 h. Thereafter, cells were harvested. To analyze the rate of anoikis, an Annexin V-fluorescein isothiocyanate (FITC) and propidium iodide (PI) Apoptosis Kit (Beyotime) was used following by the manufacturer’s instructions. Flow cytometry was used to detect anoikis cells using a FACSort system (BD Biosciences), the results of which were analyzed by CellQuest software.

### Luciferase activity assay

1.0 × 10^4^ cells were cultured in triplicate in 48-well plates for 24 h. Then, 100 ng of luciferase reporter plasmid or the control-luciferase plasmid, plus 1 ng of pRL-TK *Renilla* plasmid (Promega) were transfected into cell lines using the Lipofectamine 3000 reagent (Invitrogen), according to the manufacturer’s instructions. Luciferase signals were measured at 24 h after transfection using a Dual Luciferase Reporter Assay Kit (Promega).

### Immunofluorescence assay

Cells were plated at a density of 8000 cells/well in a 24-well plate and then washed with phosphate buffered saline (PBS), fixed with 4% paraformaldehyde treatment for 30 min, permeabilized in 0.5% Triton X-100 for 15 min, and incubated with 5% bovine serum albumin (BSA) for 30 min. Subsequently, cells were incubated with the antibodies in 5% BSA for 1 h at room temperature [rabbit anti-FOXO1 (Cell Signaling Technology)]. Afterward, cells were incubated with secondary antibodies for 1 h [goat anti-rabbit Alexa Fluor 488 (Cell Signaling Technology)]. After washing the cells with PBS, nuclei were stained with 4′,6-diamidino-2-phenylindole (DAPI; Vector Laboratories, Burlingame, CA, USA), and samples were observed on a fluorescence microscope (Olympus).

### Xenograft tumor model and tissue staining

BALB/c-nude mice (Female, 4–5 weeks old) were purchased and housed in barrier facilities on a 12 h light/dark cycle. These mice were randomly divided into different groups (n = 6/group), then AGS cells with or without HMMR silencing were injected into the abdominal cavity of mice through middle flank respectively. For Dox-induced HMMR silencing, mice were administered with Dox (2 mg/ml) in drinking water to induce HMMR downregulation at a day after tumor inoculation of AGS-shHMMR^DOX^ cells. An in vitro imaging was used to detect the growth of metastatic foci. After 6 weeks, the mice were sacrificed to explore peritoneal metastasis and examine the volume of ascites. IHC was then performed using anti-Ki67 antibodies. All animal studies were approved and performed by the animal institute of Sun Yat-sen University Cancer Center, and strictly followed the guidelines for the ethical review of laboratory animal welfare People’s Republic of China National Standard (GB/T 35892-2018). According to this guideline, the ethics committee specified that the maximal tumor burden no more than 10% of the body weight of animals and the average diameter less than 20 mm. During the experiment, the tumors size of mice complied with regulations.

### Statistical analysis

All statistical analyses were carried out using SPSS software version 19. 0 statistical software package. Statistical tests in this study included Student’s t-test (two-tailed). *P* < 0. 05 was considered statistically significant.

## Results

### Silencing of HMMR abrogated cell-to-cell interaction in vitro

**Fig. 1:**
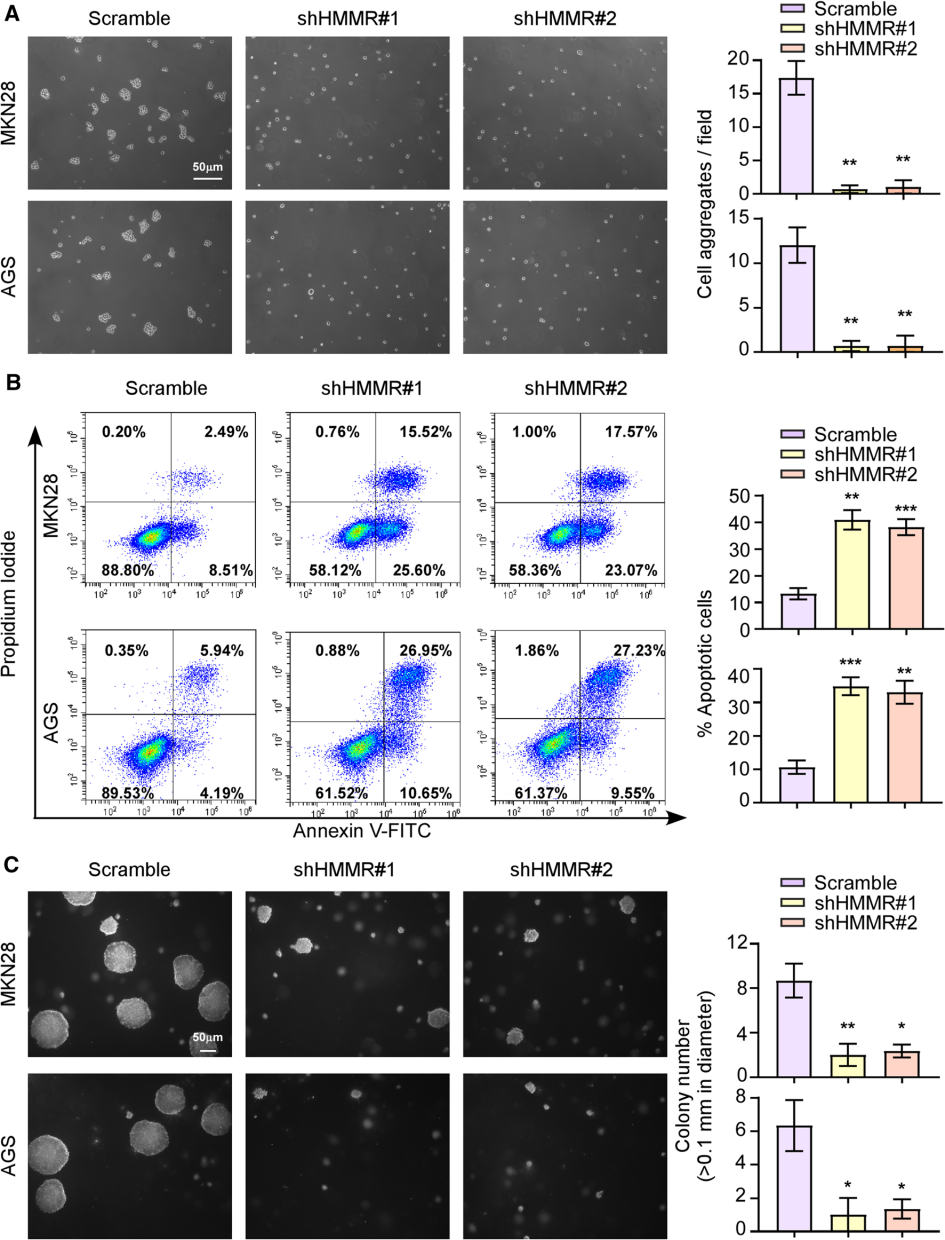
HMMR promotes cell survival and growth by increasing cell–cell interactions. **A** Representative images of cell aggregates formation of MKN28 and AGS cells with or without HMMR silencing were shown. **B** The percent of anoikis cells in indicated groups were shown. **C** Anchorage-independent growth ability was reduced in HMMR-silenced cells. Quantification of the soft agar colony formations was based on colonies > 0.1 mm in diameter. Each bar represents the mean ± SD of three independent experiments. *P* < 0.05 was considered statistically significant. *P < 0.05; **P < 0.01; ***P < 0.001

To characterize the function of HMMR in the cell-to-cell interaction, we first constructed stable AGS and MKN28 cell lines that silencing endogenous HMMR expression by using two different shRNAs. The expression level of HMMR in stable downregulated cell lines was verified by western blotting (Additional file 1: Fig. S1). Interestingly, the clustering assay indicated that silencing of HMMR significantly reduced the ability of AGS and MKN28 cells for generating multicellular clusters in suspension (Fig. 1A). To further explore the role of HMMR in cell-to-cell interaction, we practiced the anchorage-independent growth ability assay and anoikis assay. Consistently, these results showed stable depletion of HMMR abrogated the anoikis-resistant ability and the anchorage-independent growth of gastric cells (Fig. 1B, C). Collectively, these data indicated silencing of HMMR inhibited cell-to-cell interaction.

### HMMR knockdown diminished peritoneal implantation of gastric cancer

**Fig. 2:**
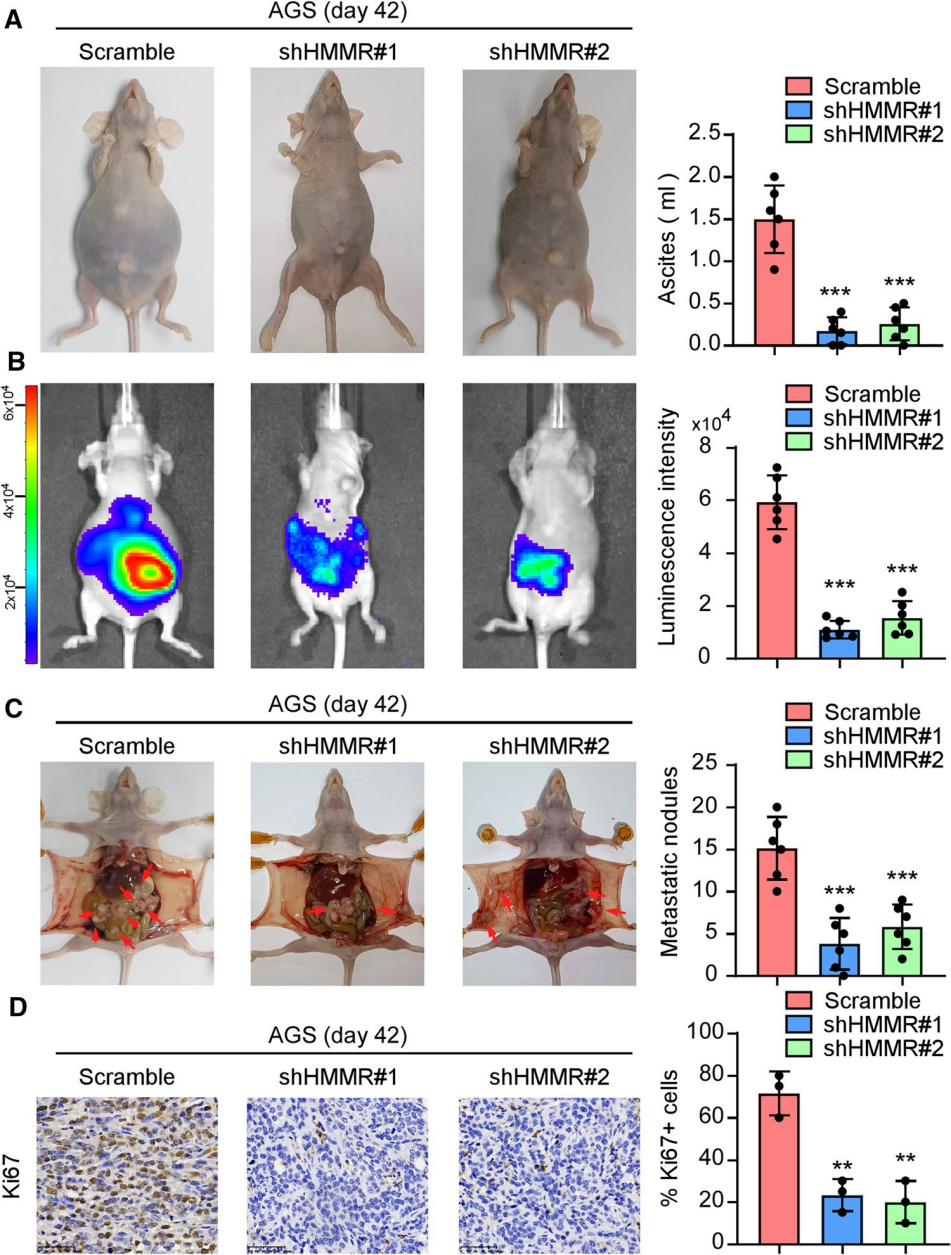
HMMR knockdown diminished peritoneal implantation of gastric cancer. **A** 1 × 10^6^ AGS cells with or without HMMR silencing were injected into the abdominal cavity of BALB/c-nu mice through middle flank (n = 6/group). Bloody ascites were examined in the mice on day 42 after i.p. inoculation in indicated groups. **B** Luminescence intensity of abdominal cavity which represent distribution of the peritoneal dissemination in indicated groups were shown. **C** Mice were killed for macroscopic examination to determine distribution of the peritoneal implantation. Silencing HMMR significantly reduced the tumor nodules in abdominal cavity. **D** Tumor nodules were obtained and IHC staining of Ki67 was performed in indicated groups. Each bar represents the mean ± SD of three independent experiments. *P* < 0.05 was considered statistically significant. *P < 0.05; **P < 0.01; ***P < 0.001

We then asked whether depleting HMMR had an impact on peritoneal implantation of gastric cancer in vivo. AGS cells with or without HMMR knockdown were injected into the abdominal cavity of BALB/c-nu mice through middle flank. Indeed, a larger volume of bloody ascites was observed in the scramble group on day 42 after i.p. inoculation of the AGS cells than in the HMMR-knockdown group (Fig. 2A). With all cells stably expressed luciferase, we observed silencing HMMR significantly reduced the bioluminescence intensity compared with the control group than others (Fig. 2B). The AGS-transplanted mice generated more tumor nodules in the peritoneal cavity and died within 50 days in the control group. On the contrary, silencing of HMMR significantly decreased the disseminated tumor nodules in the peritoneal cavity (Fig. 2C). Consistently, the staining of Ki67 in isolated tumor nodules revealed that silencing of HMMR significantly suppressed the potential of tumor proliferation (Fig. 2D). These findings confirmed the significant role of HMMR in peritoneal implantation of gastric cancer.

### HA-HMMR axis activates AKT signaling

**Fig. 3:**
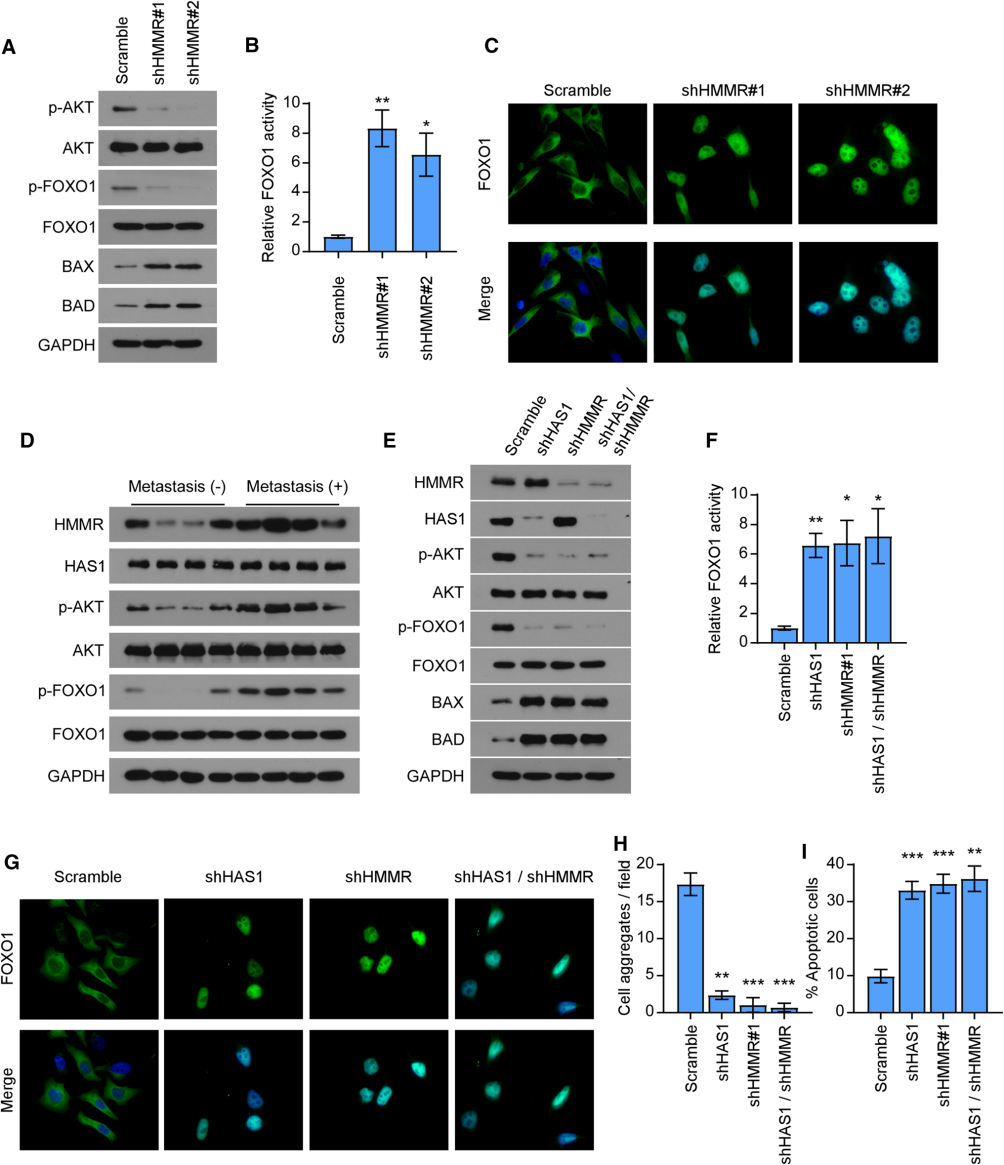
HA-HMMR axis activates AKT signaling. **A** Immunoblot analyses the expression of critical genes of AKT/FOXO1 signaling pathway (p-AKT, AKT, p-FOXO1, FOXO1, BAX, BAD) in indicated groups. GAPDH was used as a loading control. **B** Luciferase reporter assay showed the transcriptional activity of FOXO1 in control and HMMR-silencing AGS cells. **C** The immunofluorescence (IF) staining of FOXO1 was performed in indicated groups. **D** Immunoblot analysis of HMMR, HAS1, p-AKT, AKT, p-FOXO1 and FOXO1 in patients with or without peritoneum metastasis. GAPDH was used as a loading control. **E** Immunoblot analysis of HMMR, HAS1 and critical genes of AKT/FOXO1 signaling pathway (p-AKT, AKT, p-FOXO1, FOXO1, BAX, BAD) were performed in indicated groups. **F** Luciferase reporter assay showed the transcriptional activity of FOXO1 in indicated groups. **G** The immunofluorescence (IF) staining of FOXO1 was performed in indicated groups. **H** Quantification of cell aggregates formation for the indicated groups. **I** Quantification of anoikis cells in the indicated groups. Each bar represents the mean ± SD of three independent experiments. *P* < 0.05 was considered statistically significant. *P < 0.05; **P < 0.01; ***P < 0.001.

The AKT is a central element of cell-survival signaling, and a variety of intercellular contacts lead to it activation [28]. Previous studies illustrated FOXO1 exhibiting as a tumor suppressor is a vital downstream target of AKT signaling and phosphorylation of FOXO1 by AKT leads to its inactivation after nuclear to cytoplasmic translocation, which results in reducing cell growth ability [29, 30]. Considering the vital role of AKT signaling in cell–cell interaction and tumor proliferation, we next investigate whether HMMR function through activating AKT signaling. Consistent with our postulate, the results demonstrated silencing HMMR obviously inhibited the expression of p-AKT and p-FOXO1, while increased the expression of BAX and BAD (Fig. 3A). To explore whether HMMR regulates FOXO1 activity, luciferase reporter assay and immunofluorescence were performed. As shown in Fig. 3B, C, silencing HMMR significantly increased FOXO1 transcriptional activity and its nuclear import. Furthermore, we assessed the clinical relevance of HMMR and AKT-FOXO1 signaling in gastric cancer specimens. Compared with those without any metastasis, gastric cancer patients with peritoneal implantation exhibited higher expression level of HMMR and enhanced activity of AKT signaling (Fig. 3D). It is well known that HMMR was the receptor of HA, whose synthesis and secretion was promoted by HAS1. Interestingly, the expression of HAS1 was invariable in all these patients, which indicated that it’s the high expression of HMMR leads to the activation of the AKT signaling (Fig. 3D). To confirmed whether HA was indispensable in HMMR mediated AKT activation, we knockdown the HAS1 and HMMR respectively or simultaneously. We found that either knockdown HAS1 or HMMR alone did suppress AKT signaling, while promote FOXO1 nuclear translocation and increased its activity, which indicated both HA and HMMR were necessary for the activation of AKT signaling (Fig. 3E–G). Consistently, either silencing HMMR or HAS1 significantly reduced the cell aggregation formation and increased the anoikis of gastric cancer cells (Fig. 3H, I), which suggested either HA or HMMR was necessary for cell–cell interaction of gastric cancer. These findings further revealed that HMMR promoted cell–cell interaction via activating AKT signaling, which need a prerequisite for the presence of HA.

### HA is prerequisite for HMMR-mediated peritoneal implantation

**Fig. 4:**
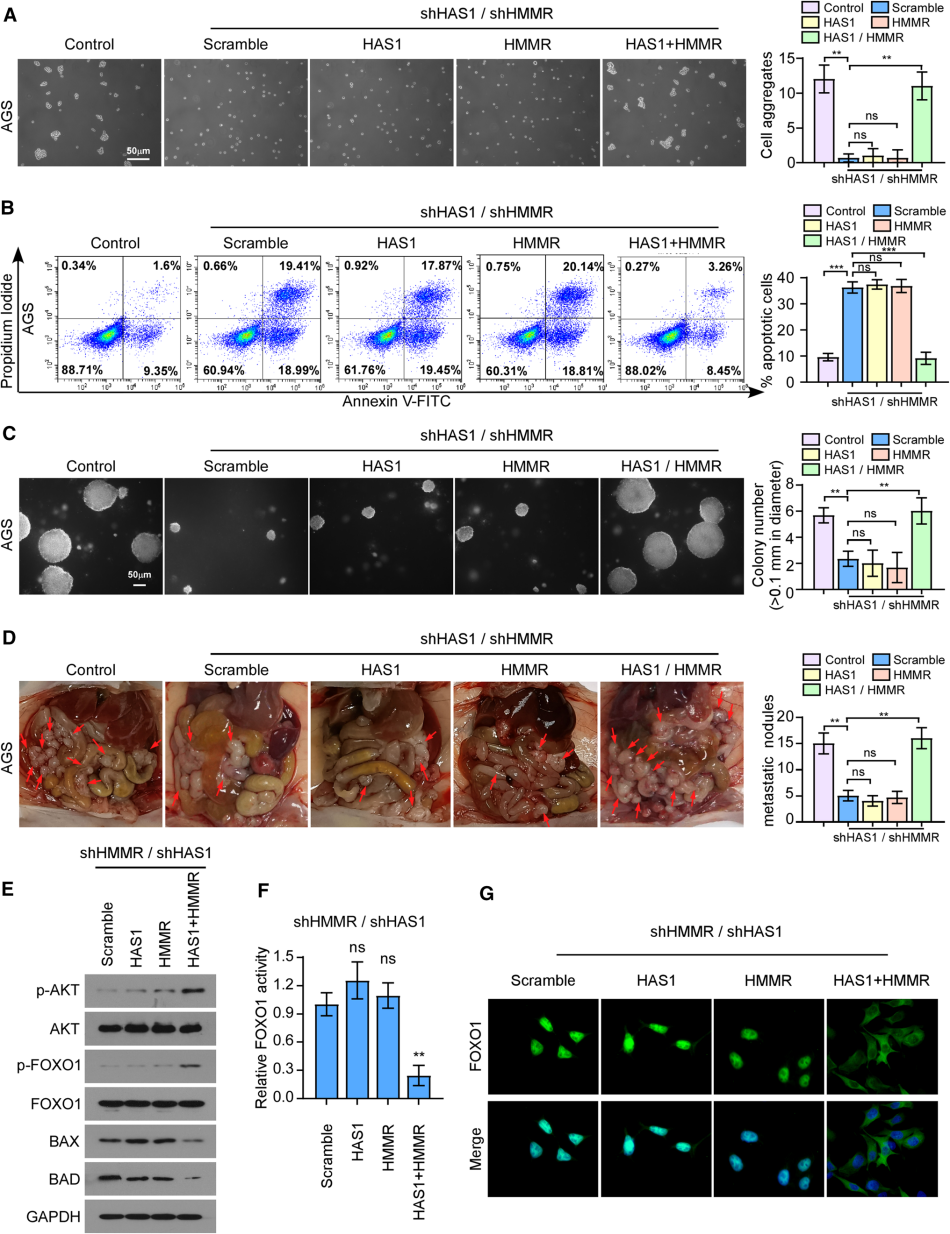
HA is prerequisite for HMMR-mediated peritoneal implantation. **A** Cell aggregates formation in indicated groups. **B** The percent of anoikis cells in indicated groups were shown. **C** Representative images of the anchorage-independent growth formations in indicated groups were shown. Quantification of the formations was based on colonies > 0. 1 mm in diameter. **D** Distribution of the peritoneal implantation in indicated groups were shown. The number of tumor nodules were quantified. **E** Western blotting showed the expression of critical genes of AKT/FOXO1 signaling (p-AKT, AKT, p-FOXO1, FOXO1, BAX, BAD) in indicated groups. **F** FOXO1 luciferase reporter assay showed the transcriptional activity of FOXO1 in indicated groups. **G** The immunofluorescence (IF) staining of FOXO1 was performed in indicated groups. Each bar represents the mean ± SD of three independent experiments. *P* < 0.05 was considered statistically significant. *P < 0.05; **P < 0.01; ***P < 0.001. ns, no significance

To further confirm the biological function of HA in HMMR-mediated cell–cell interactions, a serial of rescue experiments was performed by re-introducing HMMR gene and HAS1 gene respectively or simultaneously into both HMMR and HAS1 silencing cells (shHMMR + shHAS1). Western blotting was performed to assess gene expression in silencing cells treated with overexpressed plasmid (Additional file 1: Fig. S2). Then, the clustering assay, anoikis assay as well as soft agar assay were further evaluated. As expected, the results showed that the cell aggregates formation and anoikis-resistant ability were recovered only when the expression of HMMR and HAS1 were restored at the same time, which revealed that the expression of HAS1 was necessary for HMMR-mediated cell–cell interactions (Fig. 4A–C). Congruously, abdominal cavity injection of AGS cells with different HAS1 or HMMR expression also demonstrated HA was requisite for HMMR-mediated peritoneal implantation of gastric cancer (Fig. 4D). Expectedly, AKT-FOXO1 signaling was also activated only when both HMMR and HAS1 expression were restored which indicated HA is essential for HMMR mediated AKT signaling activation and nuclear transport of FOXO1 (Fig. 4E–G). These results revealed HA is prerequisite for HMMR-mediated peritoneal implantation.

### Inducible silencing of HMMR abrogates peritoneal implantation of gastric cancer

**Fig. 5:**
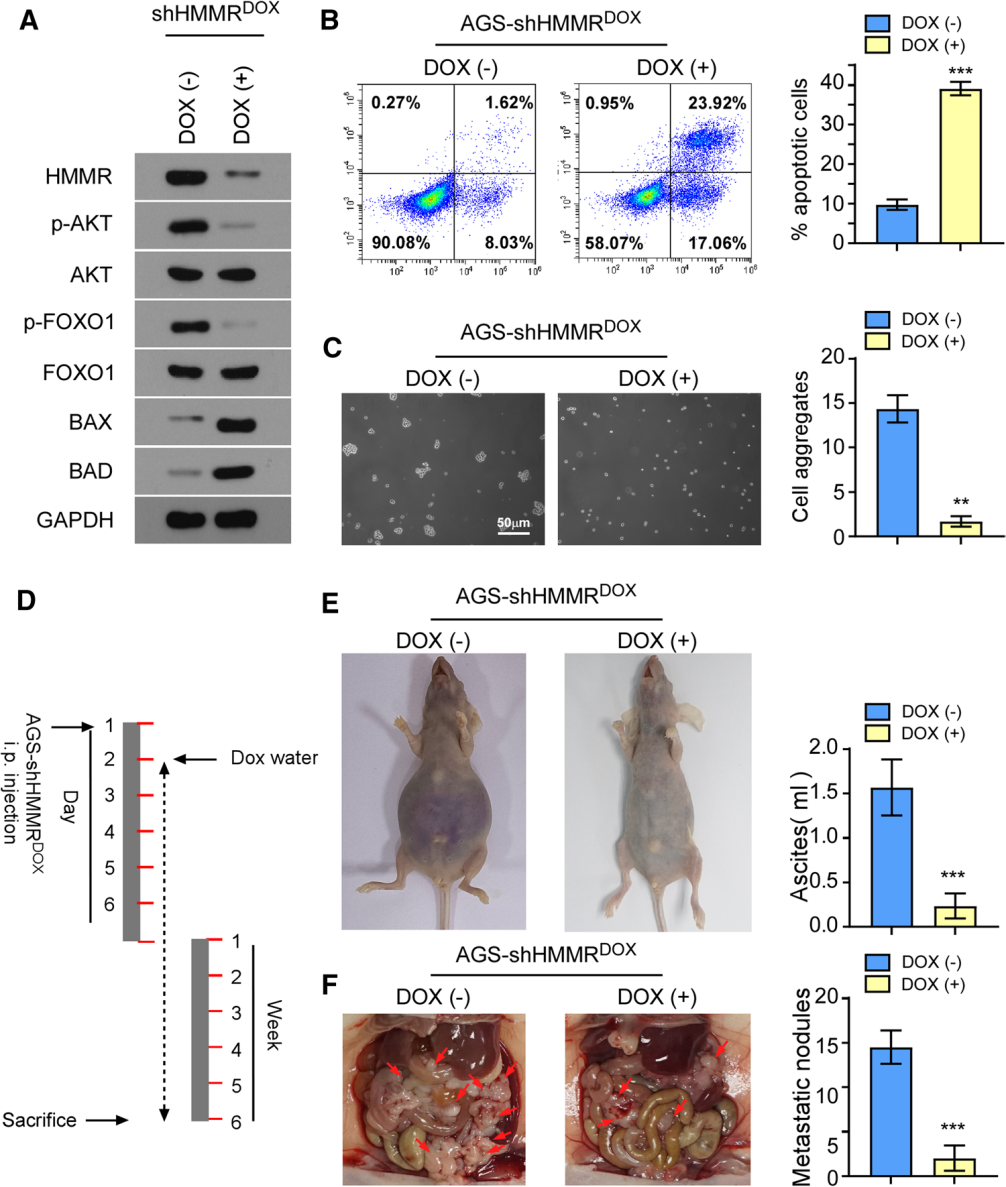
Inducible silencing of HMMR abrogates peritoneal implantation of gastric cancer. **A** Immunoblot analysis of HMMR and critical genes of AKT/FOXO1 signaling pathway (p-AKT, AKT, p-FOXO1, FOXO1, BAX, BAD) were performed in indicated groups. **B** The percent of anoikis cells in indicated groups were shown. **C** Representative images of cell clustering formations in indicated groups were shown. **D** Scheme of Dox induced mouse model. Dox-induced silencing of HMMR was performed the day after i.p inoculation. **E** Bloody ascites were observed 6 weeks after i.p inoculation. **F** Mice were sacrificed to examine the peritoneal dissemination 6 weeks after i.p inoculation. The number of tumor nodules were quantified. Each bar represents the mean ± SD of three independent experiments. P < 0.05 was considered statistically significant. *P < 0.05; **P < 0.01; ***P < 0.001

Finally, we assessed whether targeting HMMR might effectively abrogate peritoneal implantation of gastric cancer. We employed a doxycycline (Dox)-induced downregulation system to conditionally silencing HMMR expression in AGS cells. Notably, we identified that inducible silencing system has significant suppressive effect on the activation of AKT signaling (Fig. 5A). Strikingly, anoikis assay and cell aggregation formation assay indicated that depletion of HMMR reduced the anoikis resistant ability and abrogated the cell aggregation formation (Fig. 5B, C).

The therapeutic potential of HMMR targeting was further evaluated in vivo by using xenograft models. Mice were randomly divided into two groups (n = 6/group) and were intraperitoneal injected with AGS-shHMMR^Dox^. The day after inoculation, one group of mice were fed with drinking water containing Dox to induce HMMR downregulation in tumors (Fig. 5D). With the observation of tumor growth, Dox-induced silencing of HMMR potently reduced the ascites and peritoneal implantation of AGS-shHMMR^Dox^, which suggested that the abrogate in HMMR potently reduced the peritoneal implantation of gastric cancer cells (Fig. 5E, F).

## Discussion

Gastric cancer is one of the most aggressive malignancies ranking the fourth leading cause of cancer-related death around the whole world which is still a global public health threaten. Despite the therapeutic advancements reported and the great efforts paid out against this enemy, the prognosis of gastric cancer patients remains extremely gloomy. About 50% of gastric cancer patients were subjected to relapse and metastasis after radical resection, especially with a high frequent metastasis in peritoneum [31], whose median survival time was less than 3 months [6]. Although peritoneal metastasis is still a nonnegligible cause of cancer-related death in gastric cancer, but unfortunately, there is still lack of effective indicator markers and therapy method for these patients. Therefore, it is of great importance to identify efficient prognostic markers and therapeutic targets. Here, we revealed HMMR promotes peritoneal implantation of gastric cancer, which demonstrated HMMR might become a predictor and potential therapeutic target in gastric cancer patients with peritoneal metastasis.

Peritoneal metastasis is a complex process involving multiple steps and factors in which cancer cells colonization is the rate-limiting step controlling the metastatic efficiency [32]. Cell–cell interaction can enhance the adhesion of single cancer cell to form clusters thus playing a vital role in the distant colonization and metastatic growth [33]. The strong cell–cell contacts which are considered to protect clusters from anoikis endow the cancer cells with dissemination capacity [34, 35]. This kind of survival advantage along with colony-forming potential of cancer cell clusters finally lead to distant colonization and metastatic proliferation. Excitedly, the tumor cells clusters were detected in various cancers including colorectal cancer, lung cancer, breast cancer and proved to be the main source of metastasis. Aceto et al. revealed the metastatic capacity of CTCs clusters was significantly higher than single tumor cell thus giving the great contribution to breast cancer metastasis [12]. Consistently, Cheung et al. also demonstrated most of breast cancer metastases arose from tumor cell clusters [36]. This similar phenomenon also revealed in colorectal carcinoma, which tumor cell clusters were efficient initiators of peritoneal metastasis [19]. In renal cell cancer, CTCs clusters was associated with the presence of lung metastases [37]. The presence of tumor cells clusters also can be used as adverse prognostic biomarker of many cancers. Although previous studies reported that multicellular gastric cancer cells could contribute to cancer spread [38], the detailed mechanism of clusters to overcome environmental stress and initiate this colonization in peritoneum is not well known. In this study, we demonstrated HMMR could increase the cluster formation through cell–cell contacts thus leading peritoneal metastasis of gastric cancer.

HA acting as a major component of ECM have been found upregulated in various cancers. Consistently, a HA synthases HAS1 was proved to elevate in a number of tumors. HMMR, also known as RHAMM, is one of the main HA receptors also plays vital role in cancer progression. The binding of HA to its receptor HMMR can activate a series of downstream intracellular pathways, thus promoting cancer cell proliferation and invasion. Zhang et al. revealed HA-HMMR axis exhibited as a critical regulator of chemoresistance in gastric cancer via activation of TGFβ/Smad2 signaling pathway [27]. Silencing HMMR was also reported to attenuating ERK expression and phosphorylation, acting as an oncogene to maintaining the stemness and tumorigenicity of glioblastoma cell [39]. Therefore, targeting HA and its receptor interactions may identify promising therapeutic approaches in cancer treatment. Here, we demonstrated HA-HMMR signaling could increase the cell–cell interactions via activation of AKT-FOXO1 pathway, thus finally leading to distant metastasis of gastric cancer. It may pave a promising road for the early diagnosis and targeted therapy for gastric cancer. The limitation of this study is detailed molecular mechanism by which HMMR activates AKT signaling in gastric cancer remains to be illuminated, which will be the main subject of our next research.

## Conclusion

In summary, the results of the present study revealed through contacting with HA, HMMR exhibited as an oncogene to promote gastric cancer peritoneal metastasis by enhancing the cell–cell interactions and activation of AKT-FOXO1 signaling. Collectively, our findings highlighted the pro-metastatic role of HMMR, identifying HMMR as a novel prognostic biomarker and potential therapeutic target for gastric cancer.

## Supplementary Information

**Additional file 1.**

**Additional file 2.**

## Data Availability

All data generated or analysed during this study are included in this article and its additional information files.

## References

[1] Smyth EC (2020). Gastric cancer Lancet.

[2] Chen W (2016). Cancer statistics in China, 2015. CA Cancer J Clin.

[3] Catalano V (2005). Gastric cancer. Crit Rev Oncol Hematol.

[4] Zhong J, Chen Y, Wang LJ (2016). Emerging molecular basis of hematogenous metastasis in gastric cancer. World J Gastroenterol.

[5] Thrumurthy SG (2015). Does surgery have a role in managing incurable gastric cancer?. Nat Rev Clin Oncol.

[6] Riihimaki M (2016). Metastatic spread in patients with gastric cancer. Oncotarget.

[7] Motohara T (2019). An evolving story of the metastatic voyage of ovarian cancer cells: cellular and molecular orchestration of the adipose-rich metastatic microenvironment. Oncogene.

[8] Hippo Y (2001). Differential gene expression profiles of scirrhous gastric cancer cells with high metastatic potential to peritoneum or lymph nodes. Cancer Res.

[9] Guadamillas MC, Cerezo A, Del Pozo MA (2011). Overcoming anoikis–pathways to anchorage-independent growth in cancer. J Cell Sci.

[10] Gava F (2018). Gap junctions contribute to anchorage-independent clustering of breast cancer cells. BMC Cancer.

[11] van Dalum G, Holland L, Terstappen LW (2012). Metastasis and circulating tumor cells. EJIFCC.

[12] Aceto N (2014). Circulating tumor cell clusters are oligoclonal precursors of breast cancer metastasis. Cell.

[13] Dong H (2019). GLI1 activation by non-classical pathway integrin alphavbeta3/ERK1/2 maintains stem cell-like phenotype of multicellular aggregates in gastric cancer peritoneal metastasis. Cell Death Dis.

[14] Thanh Huong P (2020). Emerging role of circulating tumor cells in gastric cancer. Cancers (Basel).

[15] Kim MY (2009). Tumor self-seeding by circulating cancer cells. Cell.

[16] Gkountela S (2019). Circulating tumor cell clustering shapes DNA methylation to enable metastasis seeding. Cell.

[17] Hamilton G, Rath B (2017). Circulating tumor cell interactions with macrophages: implications for biology and treatment. Transl Lung Cancer Res.

[18] Ishii Y (2000). Integrin alpha6beta4 as a suppressor and a predictive marker for peritoneal dissemination in human gastric cancer. Gastroenterology.

[19] Zajac O (2018). Tumour spheres with inverted polarity drive the formation of peritoneal metastases in patients with hypermethylated colorectal carcinomas. Nat Cell Biol.

[20] Bayer IS (2020). Hyaluronic acid and controlled release: a review. Molecules.

[21] Morera DS (2017). Hyaluronic acid family in bladder cancer: potential prognostic biomarkers and therapeutic targets. Br J Cancer.

[22] Sun S (2020). Prognostic implications of stromal hyaluronic acid protein expression in resected oropharyngeal and oral cavity cancers. Korean J Intern Med.

[23] Hardwick C (1992). Molecular cloning of a novel hyaluronan receptor that mediates tumor cell motility. J Cell Biol.

[24] Collis L (1998). Rapid hyaluronan uptake is associated with enhanced motility: implications for an intracellular mode of action. FEBS Lett.

[25] Misra S (2015). Interactions between hyaluronan and its receptors (CD44, RHAMM) regulate the activities of inflammation and cancer. Front Immunol.

[26] Tang YP (2021). Systematic analysis of the clinical significance of hyaluronan-mediated motility receptor in colorectal cancer. Front Mol Biosci.

[27] Zhang H (2019). Hyaluronan-mediated motility receptor confers resistance to chemotherapy via TGFbeta/Smad2-induced epithelial–mesenchymal transition in gastric cancer. FASEB J.

[28] Hofmann C (2007). Cell–cell contacts prevent anoikis in primary human colonic epithelial cells. Gastroenterology.

[29] Tang ED (1999). Negative regulation of the forkhead transcription factor FKHR by Akt. J Biol Chem.

[30] Brunet A (1999). Akt promotes cell survival by phosphorylating and inhibiting a Forkhead transcription factor. Cell.

[31] Spychala A, Murawa D, Korski K (2011). The clinical importance of micrometastases within the lymphatic system in patients after total gastrectomy. Rep Pract Oncol Radiother.

[32] van Zijl F, Krupitza G, Mikulits W (2011). Initial steps of metastasis: cell invasion and endothelial transmigration. Mutat Res.

[33] Schuster E (2021). Better together: circulating tumor cell clustering in metastatic cancer. Trends Cancer.

[34] Sakamoto S, Kyprianou N (2010). Targeting anoikis resistance in prostate cancer metastasis. Mol Aspects Med.

[35] Simpson CD, Anyiwe K, Schimmer AD (2008). Anoikis resistance and tumor metastasis. Cancer Lett.

[36] Cheung KJ (2016). Polyclonal breast cancer metastases arise from collective dissemination of keratin 14-expressing tumor cell clusters. Proc Natl Acad Sci USA.

[37] Rossi E (2012). Dynamic changes of live/apoptotic circulating tumour cells as predictive marker of response to sunitinib in metastatic renal cancer. Br J Cancer.

[38] Mayer B (2001). Multicellular gastric cancer spheroids recapitulate growth pattern and differentiation phenotype of human gastric carcinomas. Gastroenterology.

[39] Tilghman J (2014). HMMR maintains the stemness and tumorigenicity of glioblastoma stem-like cells. Cancer Res.

