# Supplementary material for: HMMR promotes peritoneal implantation of gastric cancer by increasing cell–cell interactions
Source: Discov Oncol. 2022 Aug 24;13:81. doi: 10.1007/s12672-022-00543-9 (PMC9402864; doi:10.1007/s12672-022-00543-9)
Supplement: Supplementary file 1 — Additional file 1. [file 12672_2022_543_MOESM1_ESM.pdf]

SUPPLEMENTARY INFORMATION

Supplementary materials and methods

Plasmids

The shRNA sequences targeting HMMR were as follows:

shRNA#1:

GGATCCCCGCCAACTCAAATCGGAAGTATTTCAAGAGAATACTTCCGATTTGAGT

TGGCTTTTTAAGCTT;

shRNA#2:

GGATCCCCCCAGGACTAATGAACTACTAATTCAAGAGATTAGTAGTTCATTAGTC

CTGGTTTTTAAGCTT.

The shRNA sequences targeting HAS1 were as follows:

shRNA#1:

GGATCCCCGCCTATATAGGAATAACCTCTTTCAAGAGAAGAGGTTATTCCTATAT

AGGCTTTTTAAGCTT;

shRNA#2:

GGATCCCCGCATGGGTTATGCTACCAAGTTTCAAGAGAACTTGGTAGCATAACCC

ATGCTTTTTAAGCTT.

Figure S1

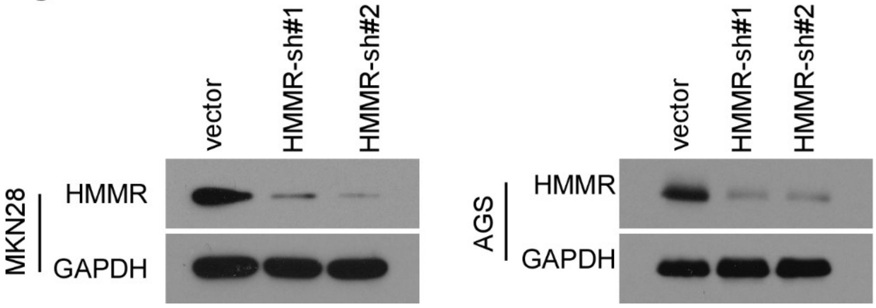

Figure S1. Gastric cancer cell lines AGS and MKN28 were stably transduced with two shRNAs. HMMR protein level was validated by western blotting analysis. GAPDH was used as a loading control.

Figure S2

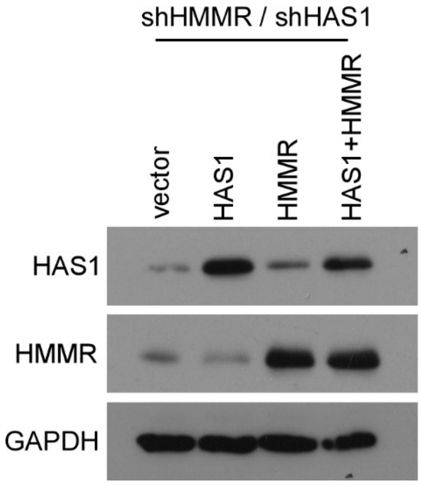

Figure S2. Western blotting validated the HMMR and HA expression in indicated groups. GAPDH was used as a loading control.
