# Supplementary figures and images for: HMMR promotes peritoneal implantation of gastric cancer by increasing cell–cell interactions
Source: Discov Oncol. 2022 Aug 24;13:81. doi: 10.1007/s12672-022-00543-9 (PMC9402864; doi:10.1007/s12672-022-00543-9)

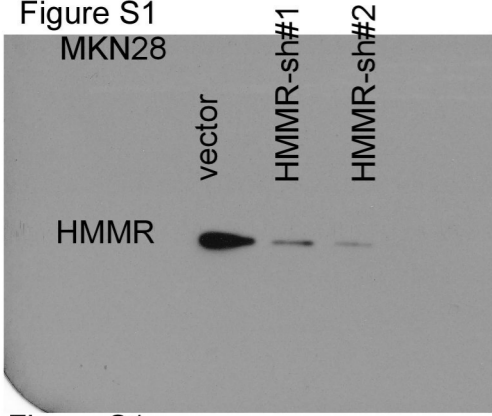

Figure S1

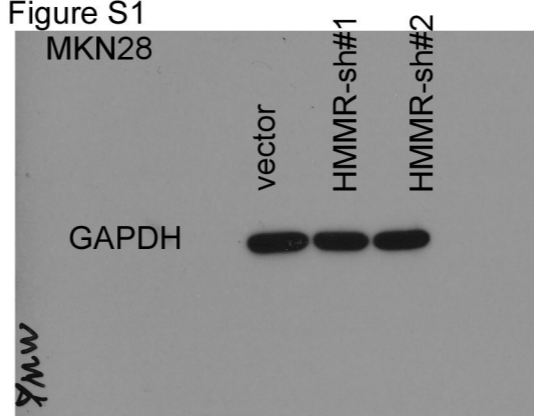

Figure S1

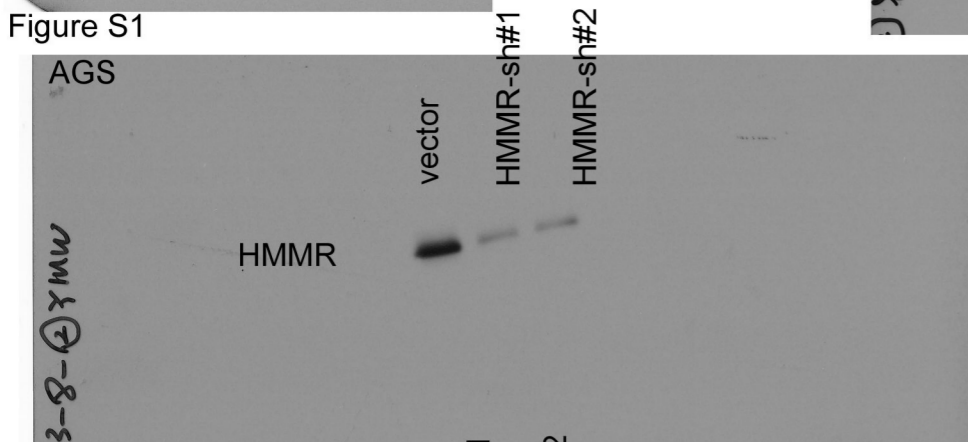

Figure S1

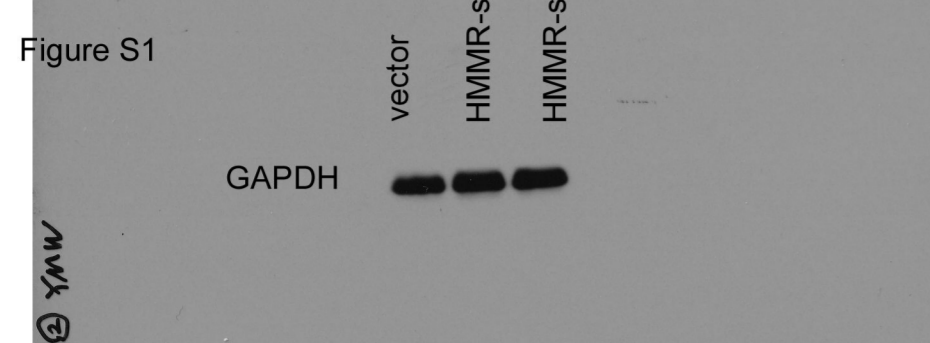

Figure 3A

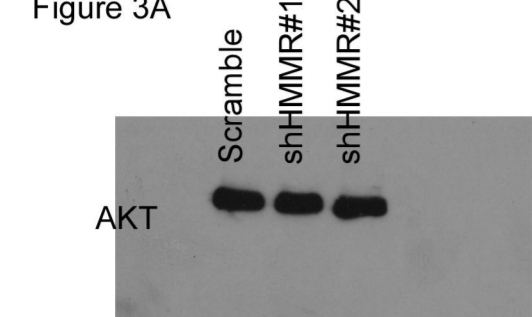

Figure 3A

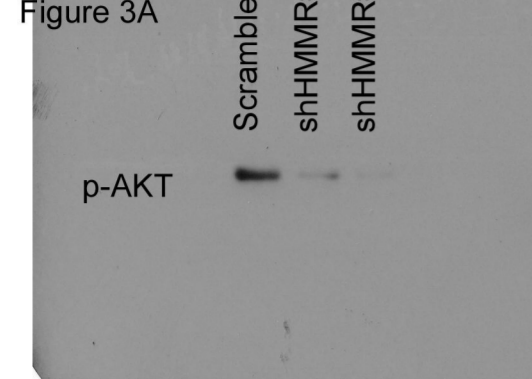

Figure 3A

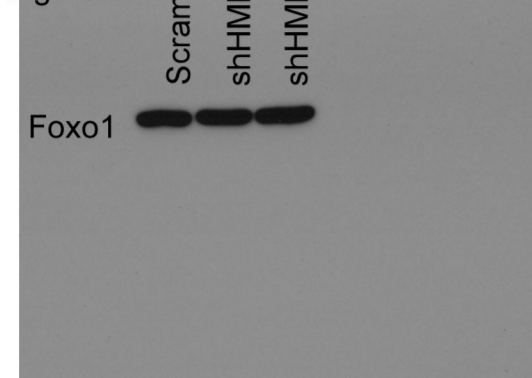

Figure 3A

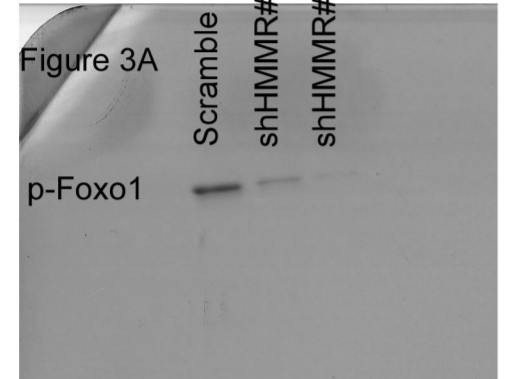

Figure 3A

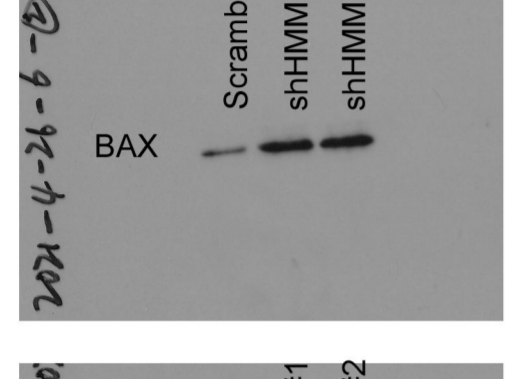

Figure 3A

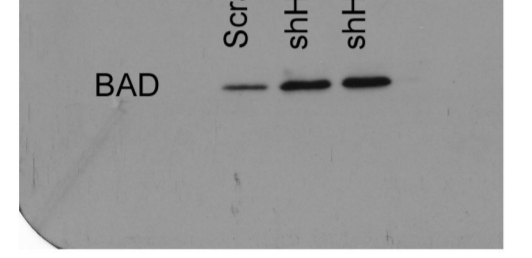

Figure 3A

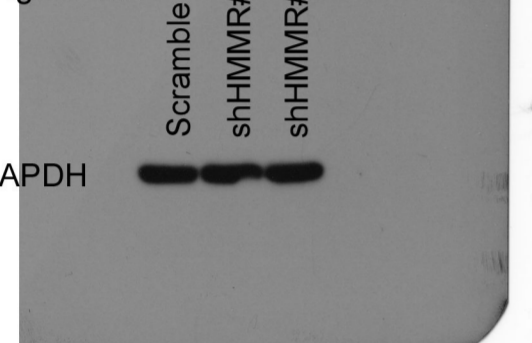

Figure 3E

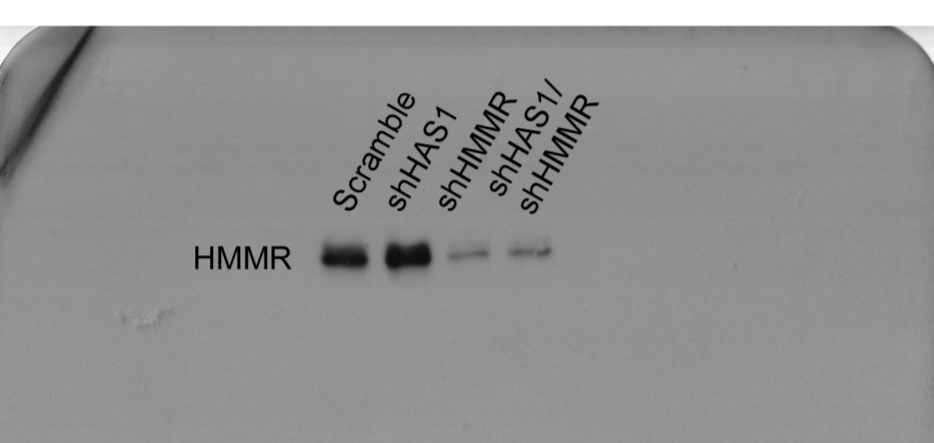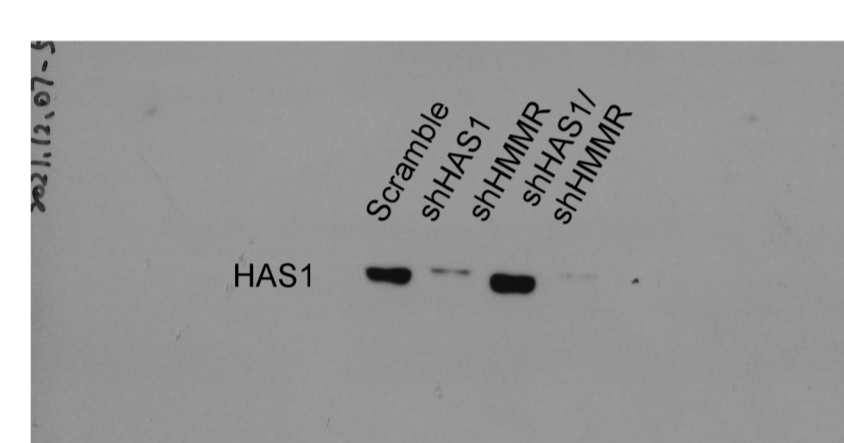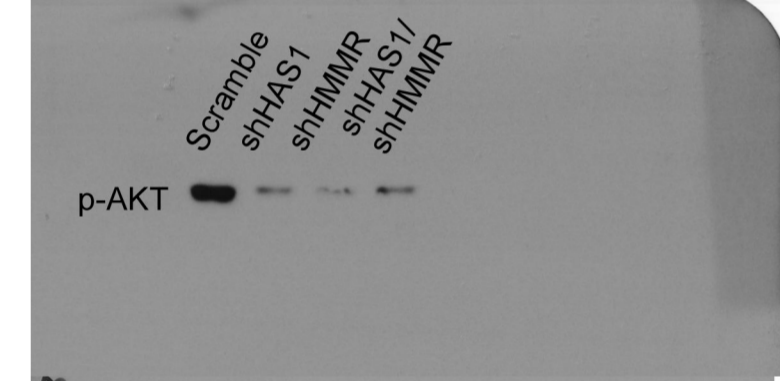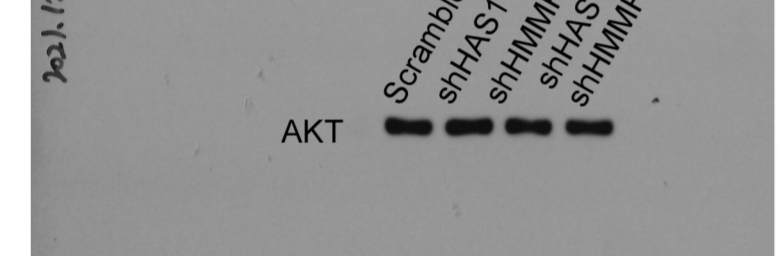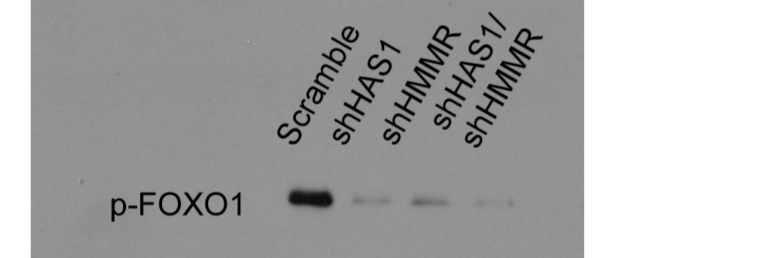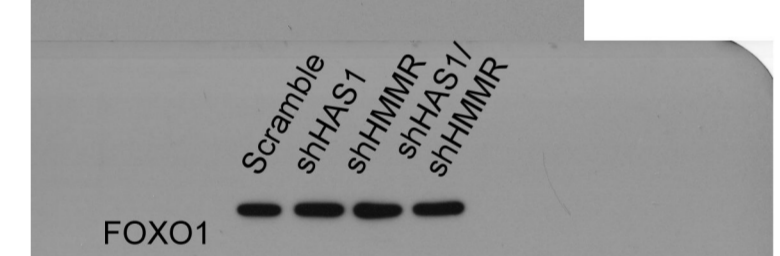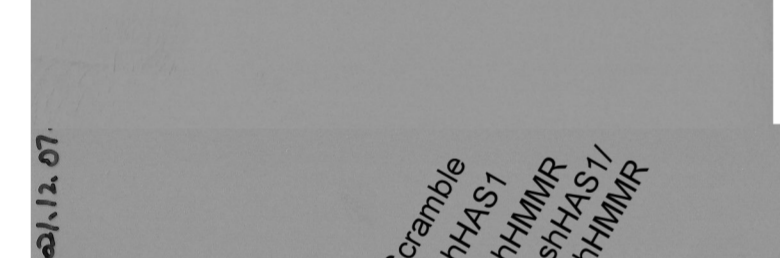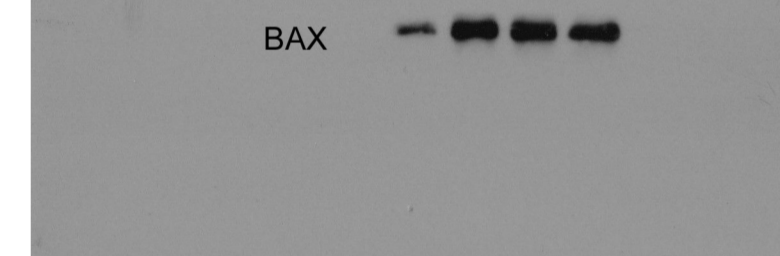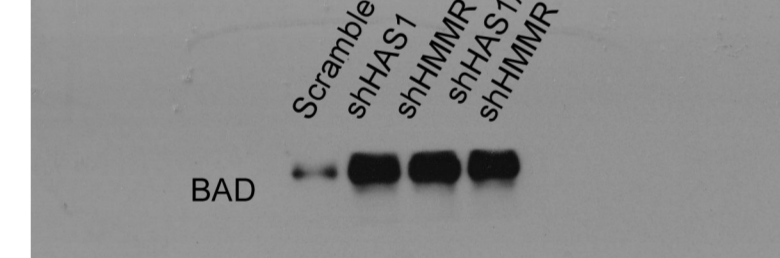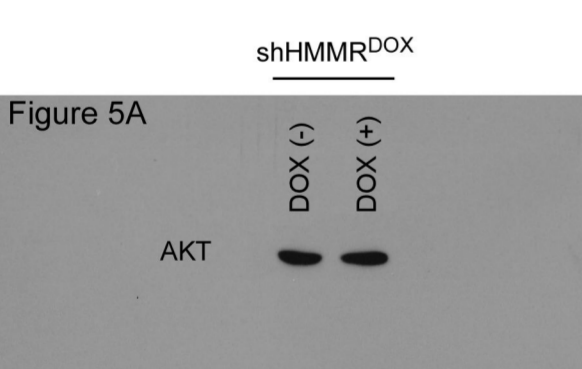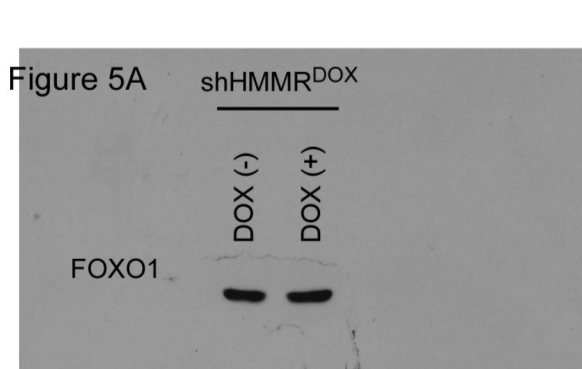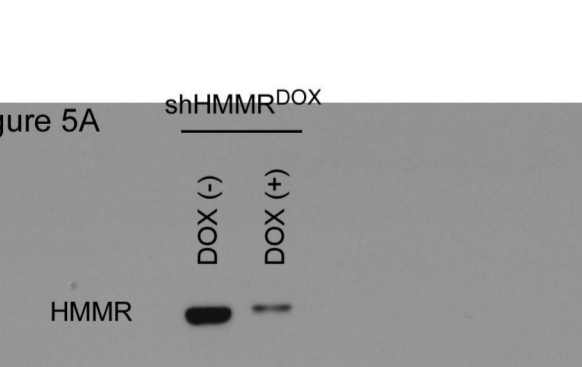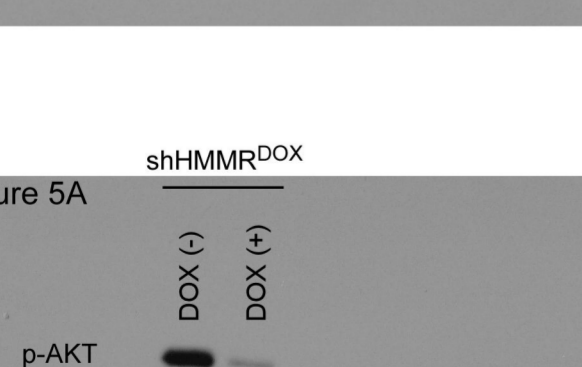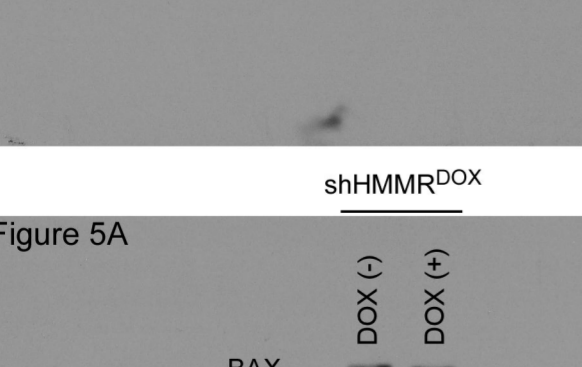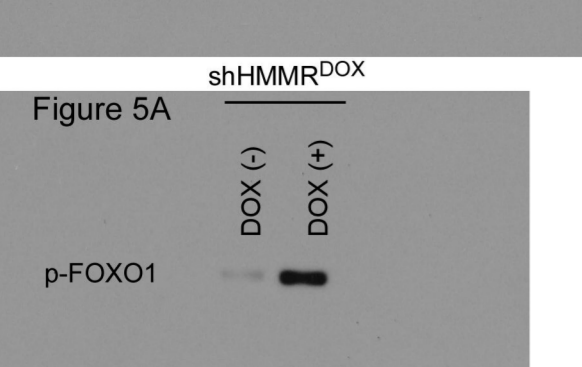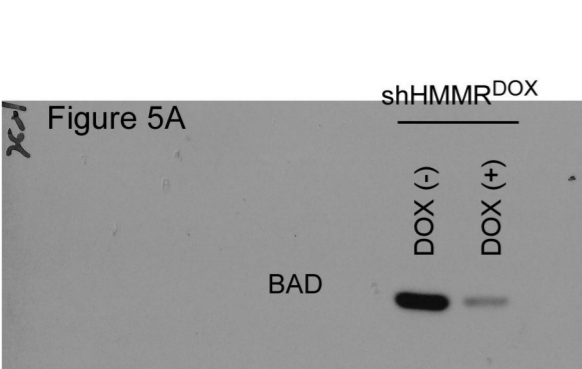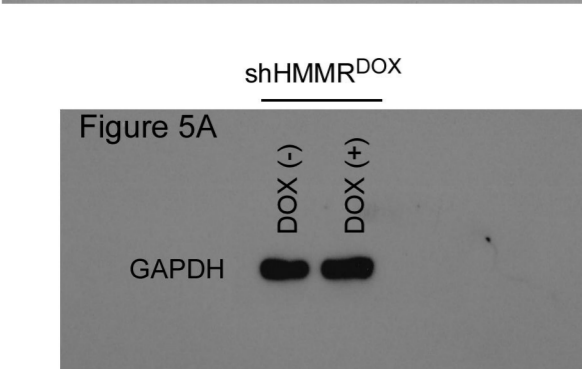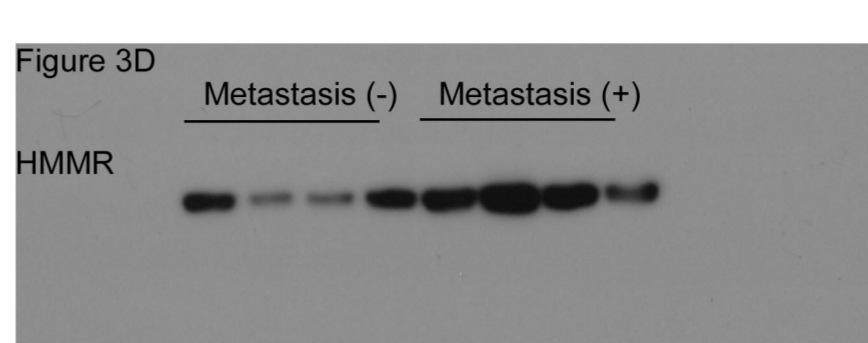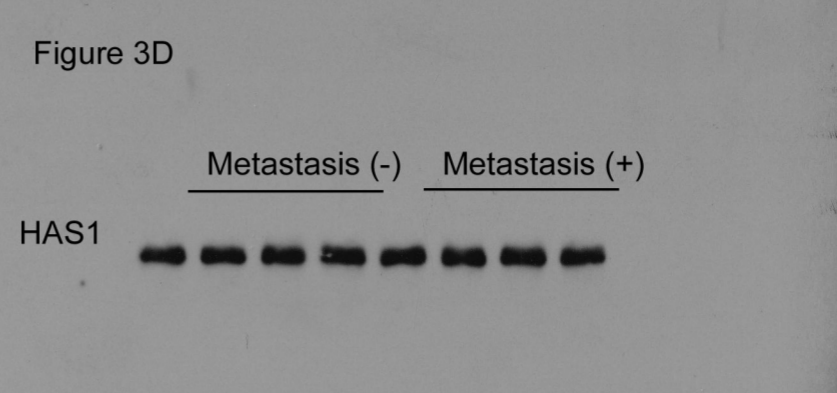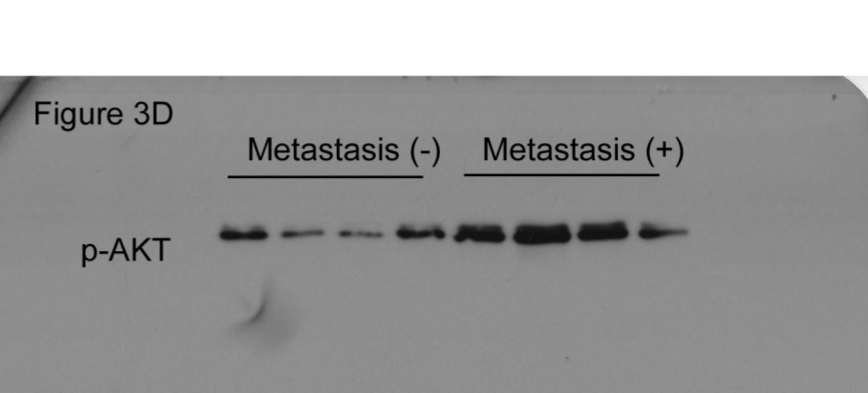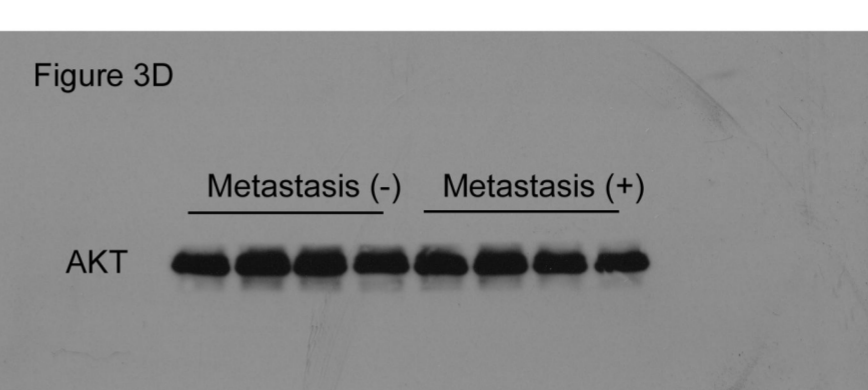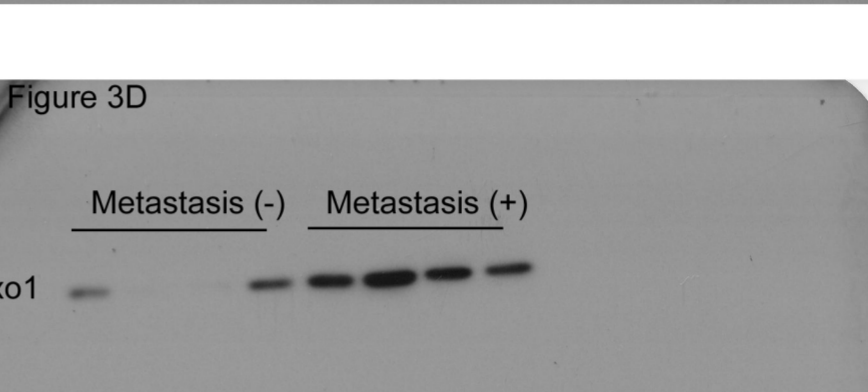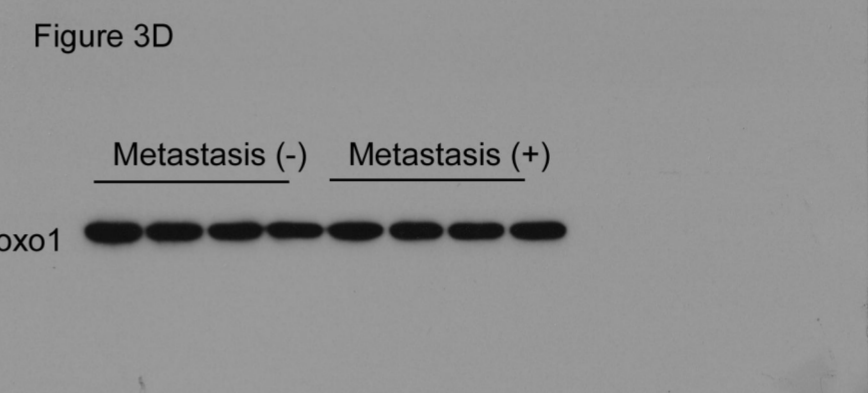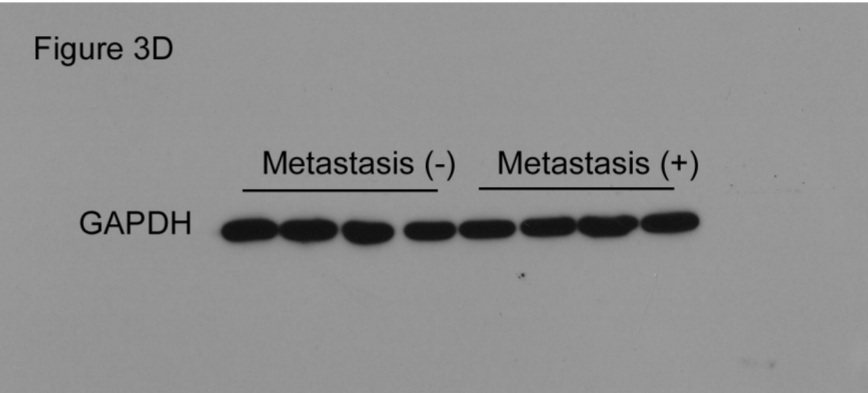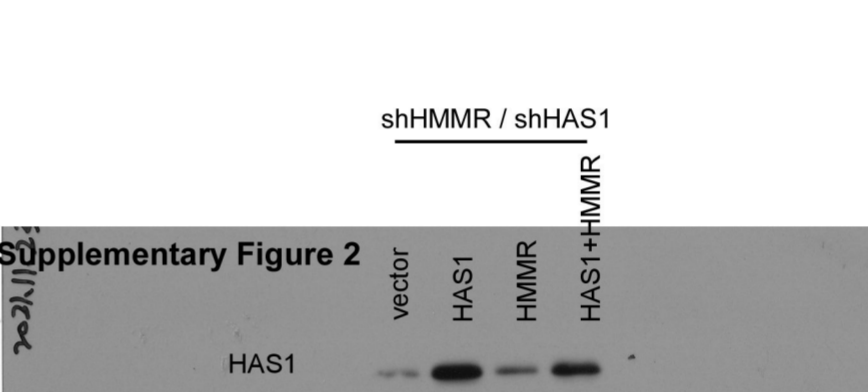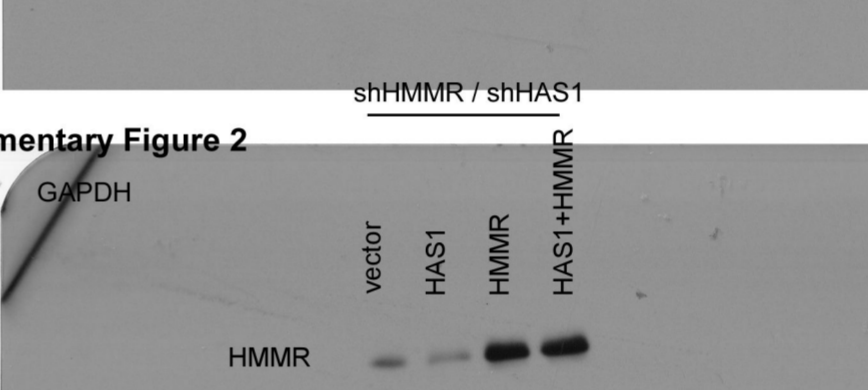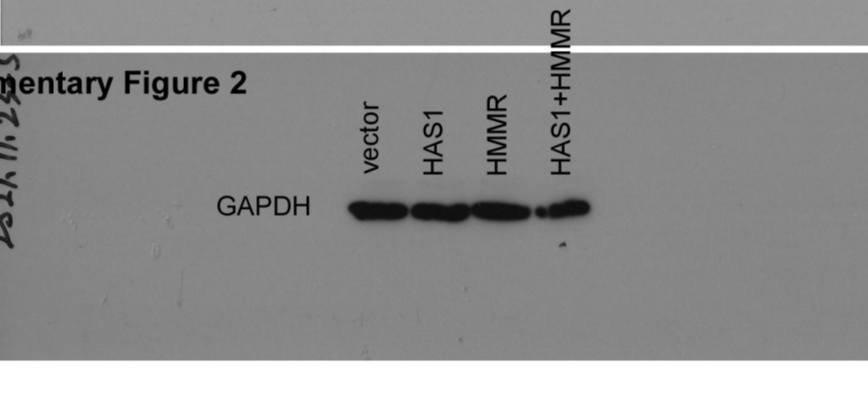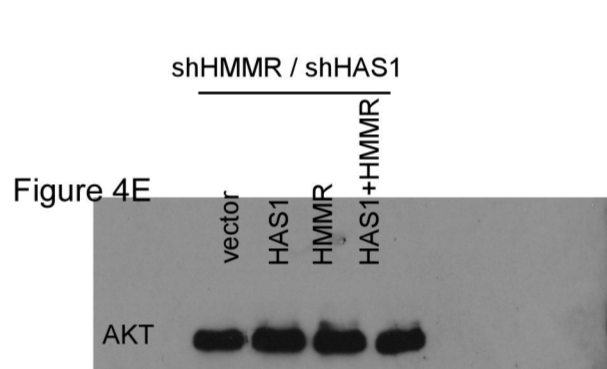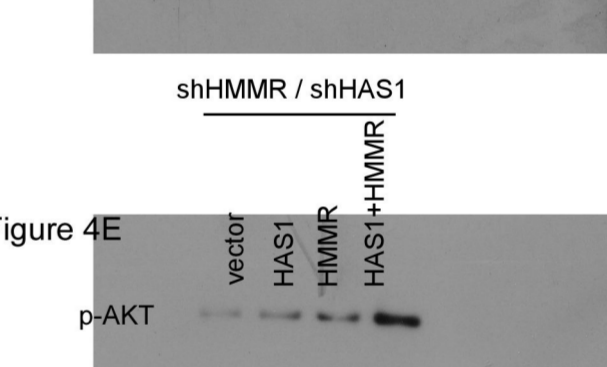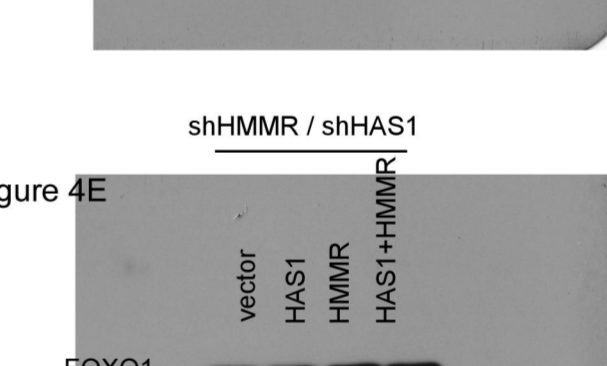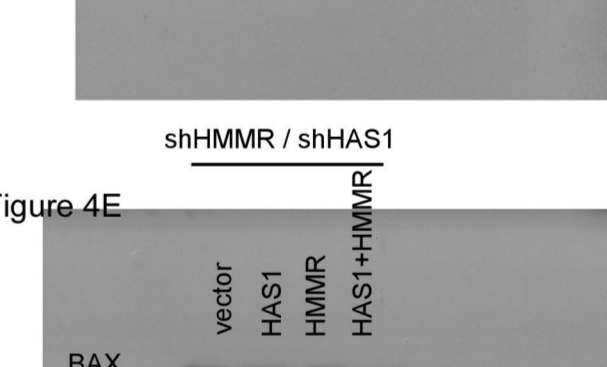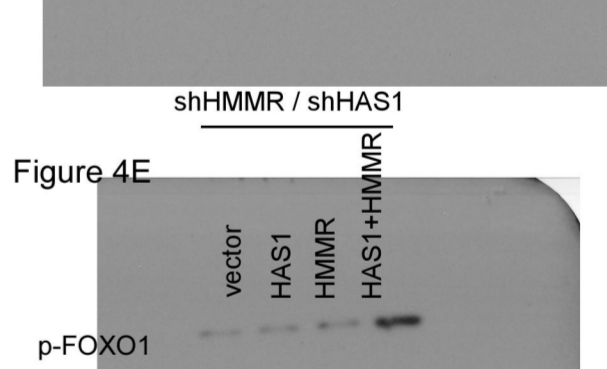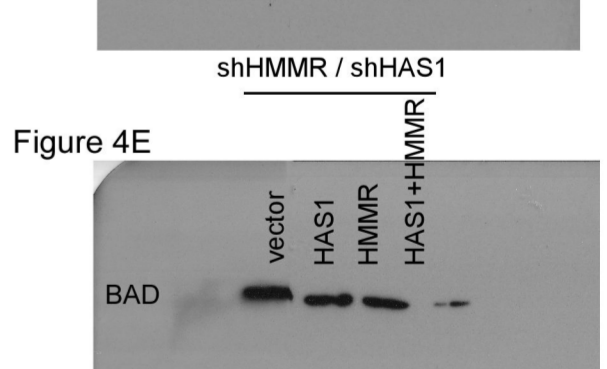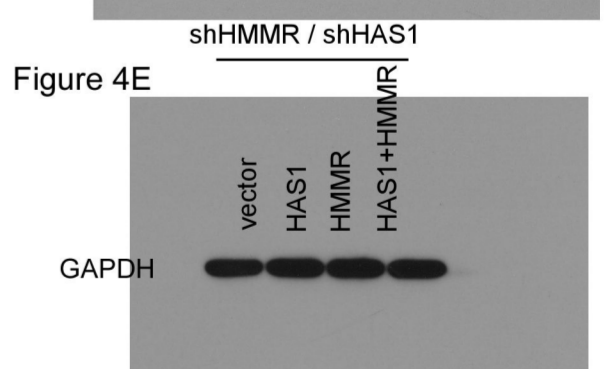

Supplement: Supplementary file 2 — Additional file 2. [file 12672_2022_543_MOESM2_ESM.pdf]
